# Case of Rupture of the Urethra, without External Wound, Occasioned by Falling across a Wooden Railing

**Published:** 1827-01

**Authors:** 

**Affiliations:** St. Thomas's Hospital.


### RUPTURE OF THE URETHRA.

*Case of Rupture of the Urethra, without external Wound, occasioned by falling across a Wooden Railing.* Treated by Mr. Travers, at St. Thomas's Hospital.

July 15th, 1826.—William Somersby, ætatis sixteen, a lad of healthy appearance, was admitted at ten p.m. with retention of urine, accompanied with severe scalding pain in the perineum, and earnest desire to empty the bladder.

On examination, the bladder was found distended and rather painful, and the perineum much swollen and very tender: from this part the cuticle was partially abraded, as it was also from the inside of both thighs, where ecchymosis to a considerable extent had taken place, producing great discoloration, in which the scrotum and penis participated. The lad walked with much difficulty, bending forward the body and separating the legs, and he complained that this exertion aggravated the pain.

On inquiry, it was ascertained that, at six P.M., while standing on some rails to take down the blinds from a window, his foot slipped, and he fell, the legs crossing the railing, and the whole weight of the body and force of the fall being concentrated upon the perineum. He immediately felt an urgent desire to void urine, but was unable to pass a single drop, and, not having passed water since the morning, the repeated ineffectual attempts produced considerable pain. The swelling and pain in the perineum came on at the same time, and, continuing to increase, he was brought to the hospital; and an attempt was made to introduce a catheter, which however passed no further than the bulb of the urethra, and there appeared to enter a cavity, in which the point readily moved in every direction: during this operation, a small quantity of blood escaped. Mr. Travers was now sent for, who on his arrival repeated the attempt with no better success; and he therefore resolved upon laying open the perineum, and endeavouring to find the continuation of the urethra, as no doubt was now entertained as to the fact of laceration having taken place.

July 16th, at half-past three A.M.—The lad being placed on a table, in the same position as in the operation of lithotomy, a deep incision was made along the line of the raphé of the perineum, and a large quantity of firmly coagulated blood removed. A catheter was then introduced at the glans penis, and the point of the instrument was seen in the wound, surrounded by the torn urethra, which appeared to have been completely divided at the bulb, one-third of its length from its termination in the spongy portion of the urethra. After a short but attentive examination, the continuation of the canal, having a clean cut edge, was found retracted about half an inch, and thrown to the left side. Into the vesical portion a silver female catheter was passed till it entered the bladder, when two pints of clear urine were drawn off. No urine appeared to have escaped previously; nor did a drop pass, notwithstanding the repeated efforts of the patient by Mr. Travers's direction, preparatory to the introduction of the catheter. The female catheter being withdrawn, a gum elastic catheter was introduced along the whole line of the urethra. Simple dressings were then applied to the wound, and retained in position by the T bandage, to which the catheter was attached. The lad was now removed to bed.

Ten A.M.—Has slept one or two hours. Has but little pain. The bladder is not distended, but there is slight tenderness of the hypogastric region on the left side. Neither fæces nor urine have been passed since the operation. The face is flushed; pulse 110, full and firm; tongue white and dry; slight thirst.

℞. Ol. Ricini ℥ ss. statim sumend.; et post duas horas repetend. nisi alvus prius responderit.

Two P.M.—The bowels have been freely relieved, and he has passed half a pint of clear urine, rather high coloured; since which the pulse has become less rapid, and his general appearance more tranquil.

For two days a little urine escaped through the wound, but on the 19th the whole passed through the catheter, and the wound was healthy and granulating. On the 22d, the catheter became partially plugged, and, notwithstanding the injection of warm water, the obstruction daily increased, and by the middle of August none of the urine escaped through it, but took its course principally through the urethra, by the side of the instrument; while a small quantity passed in drops from the wound. The catheter was, however, suffered to remain as a director to the urine, and for the purpose of obtaining a complete re-establishment of the canal of its proper dimension.

In the beginning of September, the bladder becoming rather irritable, and the wound in the perineum painful, the catheter was removed, and found coated and lined with a thick deposit of uric acid, for above two inches from the extremity which had lodged in the bladder. The removal was followed by a little hemorrhage from the urethra, which soon ceased.

September 9th.—The irritability of the bladder has subsided, and the wound is healthy and granulating; nearly one half has cicatrised. Less urine passes through the wound since the removal of the catheter.

October 11th.—The wound has now completely cicatrised, except at a pin's-point opening, through which two or three drops of urine, at the most, escape during the day. The stream of urine from the urethra is as large, and passes as freely, as before the operation.

By the application of lunar caustic to the opening, it healed in a few days; and on October 25th he was discharged.

*Case of Laceration of the Urethra without external Wound, occasioned by falling across the edge of a Boat.* Treated by Mr. GREEN, at ST. THOMAS'S HOSPITAL.

